# Three‐dimensional lung reconstructions for the localization of lung nodules to be resected during surgery

**DOI:** 10.1111/1759-7714.15131

**Published:** 2023-10-20

**Authors:** Giovanni Natale, Beatrice Leonardi, Gaetana Messina, Grazia Bergameo, Vincenzo Di Filippo, Giulia Chisari, Gabriele Raciti, Sofia Paola Lombardo, Rosa Mirra, Francesca Capasso, Francesco Leone, Davide Gerardo Pica, Alfonso Fiorelli

**Affiliations:** ^1^ Thoracic Surgery Unit, Department of Translation Medicine Università della Campania “LuigiVanvitelli” Naples Italy; ^2^ Genomics and Experimental Oncology Unit, IOM Ricerca Viagrande Italy

**Keywords:** 3D lung reconstruction, digital 3D models, lung nodule localization

## Abstract

**Background:**

The localization of lung nodules is challenging during thoracoscopy. In this study, we evaluated the use of three‐dimensional (3D) lung reconstruction for use in the operating room to guide the identification of lung nodules during thoracoscopy.

**Methods:**

This was a single‐center retrospective study. All consecutive patients undergoing thoracoscopic resection of lung nodules were included in the study. Patients were retrospectively divided into two groups based upon whether the thoracoscopic resection was performed with the assistance (3D group) or not (standard group) of 3D lung reconstruction. The operative time (minutes) to detect lung nodules was statistically compared between the two study groups in relation to the characteristics of lung nodules as size, localization, and distance from the visceral pleura.

**Results:**

Our study population consisted of 170 patients: 85 in the 3D group and 85 in the standard group. No intergroup difference differences were found regarding the characteristics and histological diagnosis of lesions. The standard group compared to the 3D group was associated with a significantly longer operative time for the detection of lesions <10 mm (13.87 ± 2.59 vs. 5.52 ± 1.01, *p* < 0.001), lesions between 10 and 20 mm (5.05 ± 0.84 vs. 3.89 ± 0.92; *p* = 0.03), lesions localized in complex segments (7.49 ± 4.25 vs. 5.11 ± 0.97; *p* < 0.001), and deep lesions (9.58 ± 4.82 vs. 5.4 ± 1.01, *p* < 0.001).

**Conclusions:**

Our 3D lung reconstruction model for use in the operating room may be an additional tool for thoracic surgeons to guide the detection of small and deep nodules during thoracoscopy. It is a noninvasive and cost saving procedure and may be widely used.

## Graphical abstract

Intraoperative localization of lung nodules is challenging during thoracoscopy. We evaluated the use of 3D lung reconstruction for use in the operating room to guide the intraoperative identification of lung nodules during thoracoscopy.

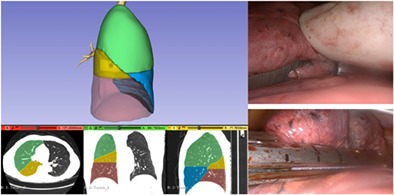

## INTRODUCTION

Lung cancer is the main cause of cancer‐related death worldwide. The increased use of low‐dose computed tomography (CT) in lung cancer screening programs allows the detection of small and multiple lung nodules. The diagnosis of these lesions remains challenging. Transthoracic needle biopsy has a limited yield rate in cases of small and deep nodule and may be complicated with pneumothorax in chronic obstructive pulmonary disease (COPD) patients.^1^ Resection by thoracoscopy is a minimally invasive strategy that provides sufficient materials for early diagnosis and molecular analysis; furthermore, it may have a curative intent in patients with lung metastases when the primary cancer and extra‐thoracic metastasis is controlled.^2^ However, the localization of lung nodules may be difficult during thoracoscopy and over the years several strategies have been proposed to facilitate this procedure such as the use of ultrasonography or electromagnetic navigation bronchoscopy, marking the nodule with hook wire, radiolabeled aggregates, microcoils, and liquid agents.^3^ However, each technique has strengths and limitations and the best strategy is therefore still debatable.

Herein, we report a novel strategy using a three‐dimensional (3D) lung reconstruction for use in the operating room to guide the identification of lung nodules during thoracoscopy.

## METHODS

### Study design

This was a single‐center retrospective study. All consecutive patients with indeterminate lung nodules who underwent thoracoscopic resection between January 2019 and April 2022 were eligible. We have been making 3D reconstructions of the lung parenchyma and nodules for mainly educational purposes since early 2021. Clinical cases undergoing 3D reconstruction are consecutive and have been compared with clinical cases prior to the use of preoperative 3D reconstruction. In this analysis, we included the clinical characteristics of patients with nodules: (1) less than 30 mm in size, (2) solid or part‐solid, and (3) without a definitive preoperative diagnosis. We excluded the data of patients with: (1) nodules more than 30 mm, (2) pure ground‐glass opacity (pGGO), (3) multiple lesions, and (4) incomplete data on the characteristics of lesions.

Patients were retrospectively divided into two groups based upon whether the thoracoscopic resection was performed with the assistance (3D group) or not (standard group) of 3D lung reconstruction. The operative time to detect lung nodules was statistically compared between the two study groups. The hypothesis of the study was that the use of 3D lung reconstruction would reduce the operating time. The results were evaluated in relation to the characteristics of lung nodules as size, localization, and distance from the visceral pleura.

All patients underwent wedge resection with frozen section analysis and based on the pathological report lobectomy with hilo‐mediastinal lymphadenectomy was performed if there were malignant cells; otherwise, the surgery was terminated. All patients gave their signed informed consent for the operation, and they were aware that their clinical data could be used anonymously for scientific purposes only. The study was approved by the local ethics committee of our hospital.

### Data collection and classification of nodules

For each patient we collected the following data: demographic data; lesion size (mm), distance of nodule from pleura (mm), location of nodule, operative time for nodule detection (minutes), histological diagnosis, conversion rate, and intraoperative complications. In all cases, the correct detection of nodule was confirmed by frozen analysis. According to Fleischner Society guidelines,^4^ a lung nodule was defined as a rounded pulmonary opacity with well‐defined or poorly defined margins, measuring <30 mm, and surrounded by pulmonary parenchyma. Based on the eighth edition of TNM classifications of lung cancer, the lesions were divided into three different subgroups as follows: ≦10 mm (T1a lesion), >10–20 mm (T1 b lesion), and >20–30 mm (T1c lesion).^5^ The lesions were classified as deep or peripheral if the distance from the visceral pleura were more or less than 10 mm, respectively.^6^ Then, the lesion was also differentiated as localized in simple or complex segment, based on the classification of Suzuki et al.^7^

### 3D lung reconstruction model

**FIGURE 1:**
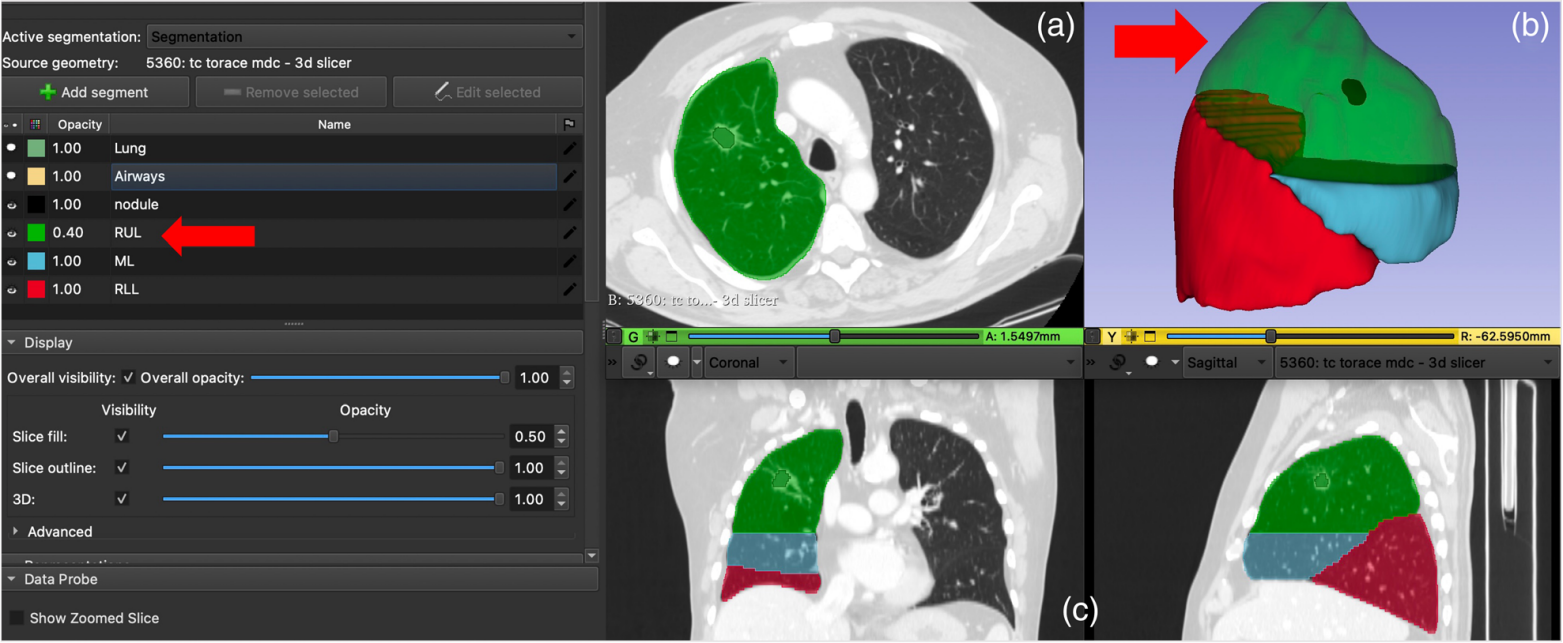
The lobes of the target lung are displayed in a three‐dimensional (3D) view in a different color (a). The target lobe was put in transparency to define the characteristics of the lesion (b). The segments of the target lobe are displayed in a different color to define the segmental localization of the nodule (c).

3D‐slicer (Brigham and Women's Hospital, Boston) free software was used to create the 3D model.^8^ This is an open‐source program, totally free and easy to use. To be used optimally it requires a good knowledge of anatomy seen on CT scan but without needing specific skills in radiology. The 3D lung reconstruction model was created step by step as follows. (1) The preoperative images of a chest CT scan acquired with a thickness less than 1.5 mm were uploaded as DICOM format in the software and displayed in multiplanar reconstruction (MPR). The lobes of the target lung were displayed in a 3D view in a different color (Figure 1a). (2) The target lobe was put in transparency in order to define the characteristics of the lesion as size, and the distance from the visceral pleura (Figure 1b). (3) A specific application of the software named as “segment editor” allowed a digital model of the target structure as the lesion and the specific segment where the lesion was located based on the different density of the structures to be separately created. At the end of the segmentation process, the anatomical structures could be moved 360 degrees and displayed in a different color to define the segmental localization of the nodule (Figure 1c). (4) The images and the video of the 3D model were then saved on standard USB devices so they could easily be uploaded to the operating room monitor and/or a standard mobile device such as a tablet, if an additional monitor during the operation was not available. They could then be reviewed in real time during the operation (Video S1).

### Operative procedure

All patients underwent thoracoscopy with an anterior approach according to Copenhagen with a 10 mm length camera incision trough the VII intercostal space along the mid‐axillary line, and 30–40 mm length incisions for working instruments through the IV or the V intercostal space along the anterior axillary line. After exploration of the pleural cavity, and the lung was determined to be free of all adhesions, the nodule was identified by finger palpation (when possible) and/or by forceps, and was then resected using a stapler in a standard approach. In the 3D group, the real‐time view of the digital 3D model guided the identification of the lesion (Video S2). The specimen was retrieved and sent for intraoperative frozen section examination. In cases where primary lung cancer was diagnosed, a standard anatomical resection of the target lobe and/or segment was performed in the same setting. If the target lesion was not detected during thoracoscopy, the anterior incision was enlarged to allow a manual palpation of the lung for the detection of a lesion.

### Statistical analysis

Data are summarized as mean and standard deviation (SD) for continuous variables, and absolute number and percentage for categorical variables. Intergroup differences were compared using mean and interquartile range (IQR), as appropriate. A *p* < 0.05 was considered statistically significant. MedCalc statistical software (version 12.3) was used for analysis.

## RESULTS

**FIGURE 2:**
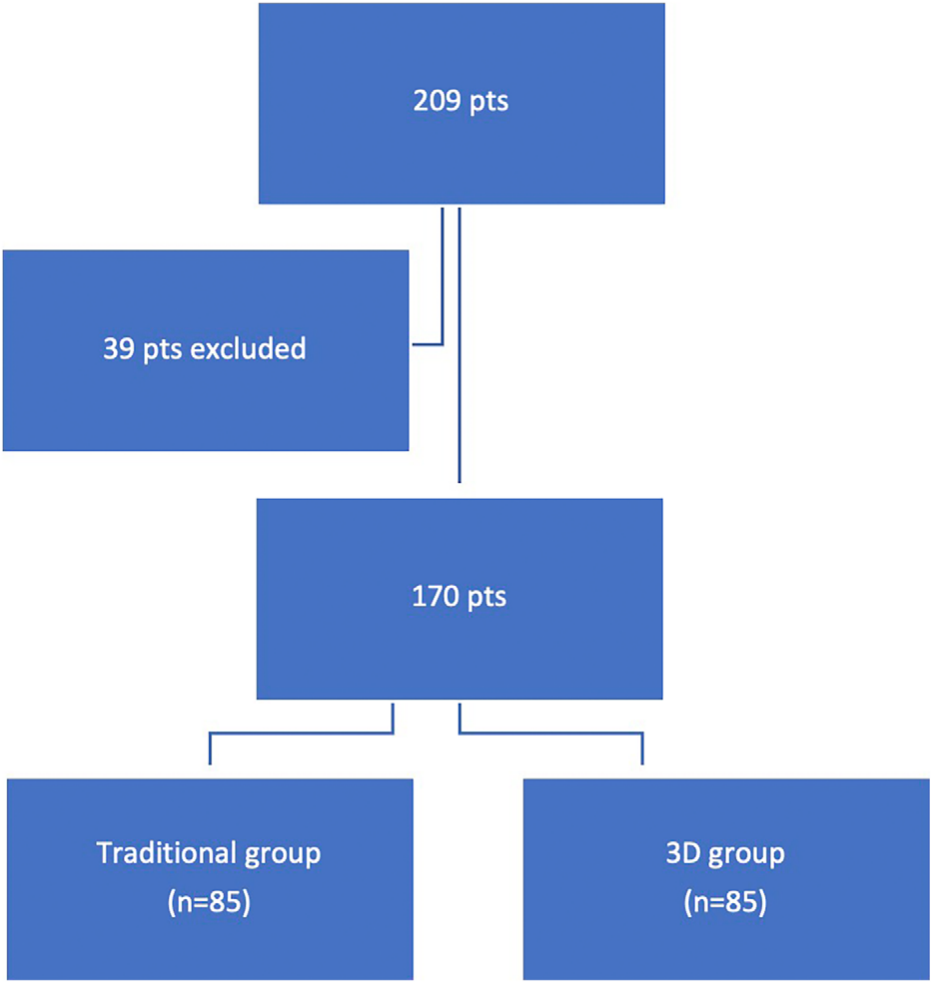
Study flow chart.

In the study period, 209 patients underwent thoracoscopic biopsy of lung lesions. Of these, the data of 39 patients were excluded due to the presence of multiple lesions (*n* = 3), GGO lesions (*n* = 17), lesions larger than 30 mm (*n* = 15), and incomplete data (*n* = 4) regarding the characteristics of nodule. Thus, our study population consisted of 170 patients; 85 patients were included in the 3D group and 85 patients were included in the standard group if (1) they were treated before the utilization of the 3D digital model in our hospital, or (2) the quality of their CT images was poor for creating a 3D model (i.e., thickness slice more than 1.5 mm). The flow chart of the study is summarized in Figure 2. In all patients but one the nodule was detected during thoracoscopy and successfully resected without intraoperative complications. The frozen section analysis diagnosed a primary lung tumor in 56 patients, who underwent lobectomy in the same setting. In the remaining 45 patients, it diagnosed a lung metastasis in 36 cases, a benign lesion in seven cases, and a nondefinitive diagnosis of cancer in 18.

As summarized in Table 1, the comparison of the standard versus the 3D group showed no significant difference regarding age (*p* = 0.45), sex (*p* = 0.59), respiratory functions as forced expiratory volume (FEV) 1% (*p* = 0.09), and diffusing capacity for carbon monoxide (DLCO%) (*p* = 0.27), standardized uptake value (SUV) (*p* = 0.69), the affected lobes, the simple (*p* = 0.61) and complex affected segments (*p* = 0.62), lesion size (*p* = 0.84), distance from visceral pleural (*p* = 0.72), histological diagnosis and conversion rate (*p* = 0.12).

**TABLE 1:** Characteristics of study population.

Variables	All patients (*n* = 170)	Standard group (*n* = 85)	3D group (*n* = 85)	*p‐*value
Age, years (mean)	67.25 ± 6.7	67.43 ± 6.62	66.9 ± 6.8	0.45
Sex (male), *n* (%)	106 (62)	51 (60)	55 (65)	0.59
FEV1 (%)	84.74 ± 13.63	85.18 ± 10.4	84 ± 16.3	0.09
DLCO (%)	90 ± 12.85	90 ± 12.2	89 ± 13.5	0.27
SUVmax	4.94 ± 3.32	7.45 ± 3.29	7.16 ± 5.8	0.69
Affected lobe				
RUL	50	28 (33)	22 (26)	0.28
ML	4	1 (1)	3 (4)	0.016
RLL	47	18 (21)	29 (34)	0.892
LUL	31	19 (22)	12 (14)	0.297
LLL	38	19 (22)	19 (22)	1
Affected simple segment	41	19 (20)	22 (23)	0.61
Right S4 + 5	4	1 (1)	3 (4)	0.9
Right S6	14	6 (7)	8 (9)	0.89
Left S4 + 5	9	5 (6)	4 (5)	0.95
Left S6	14	7 (8)	7 (8)	1
Affected complex segment	151	78 (80)	73 (77)	0.62
Right S1	19	11 (13)	8 (9)	0.79
Right S2	14	8 (9)	6 (7)	0.89
Right S3	17	9 (11)	8 (9)	0.89
Right S7	4	2 (2)	2 (2)	1
Right S8	8	3 (4)	5 (6)	0.91
Right S9	12	4 (5)	8 (9)	0.81
Right S10	9	3 (4)	6 (7)	0.87
Left S1	13	8 (9)	5 (6)	0.85
Left S2	9	6 (7)	3 (4)	0.86
Left S3	13	8 (9)	5 (6)	0.85
Left S8	7	4 (5)	3 (4)	0.95
Left S9	8	4 (5)	4 (5)	1
Left S10	9	4 (5)	5 (6)	0.95
	9	4 (5)	5 (6)	0.95
Size (mm)		16 ± 6.65	15.8 ± 6.2	0.84
≦10	48	23 (27)	25 (29)	0.88
>10 to 20	69	35 (41)	34 (40)	0.93
>20 to 30	53	27 (32)	26 (31)	0.93
Distance from the visceral pleura (mm)	9.33 ± 5.99	9.63 ± 6.11	9.3 ± 5.9	0.72
Peripheral	4.91 ± 2.2	4.88 ± 2.25	4.94 ± 2.18	0.86
Deep	15.66 ± 4.47	15.77 ± 3.65	15.54 ± 3.34	0.78
Histology, *n* (%)				
Adenocarcinoma	72	28 (33)	44 (52)	0.11
Squamous carcinoma	35	18 (21)	17 (20)	0.94
Large cell carcinoma	2	1 (1)	2 (2)	0.95
Nondefinitive diagnosis of malignancy	18	11 (22)	7 (15)	0.63
Benign lesion	7	4	3	0.49
Lung metastasis	36	23 (22)	13 (11)	0.41
Conversion rate	1	1	0	

Abbreviations: 3D, three dimensional; DLCO, diffusing capacity for carbon monoxide; FEV1, forced expiratory volume; LLL, left lower lobe; LUL, left upper lobe; ML, middle lobe; RLL, right lower lobe; RUL, right upper lobe; SUV, standardized uptake value.

### Operative time for nodule identification

The results are summarized in Table 2. The mean operative time for nodule identification was longer in the standard compared to the 3D group (7.41 ± 4.21 min vs. 5.07 ± 0.96 min, *p* < 0.001). This difference in average operating time in the two groups was especially important when we analyzed the operating time of identification of lung nodules smaller than 10 mm and those between 10 and 20 mm, as specified below.

**TABLE 2:** Operative time for detection lung lesions in relation to the characteristics of the lesions.

Operating time (min) Mean ± SD [IQR]	All patients (*n* = 170)	Standard group (*n* = 85)	3D group (*n* = 85)	*p‐*value
Operative time (min)	6.24 ± 3.05	7.41 ± 4.21 [5–11]	5.07 ± 0.96 [4–6]	<0.001
Lesions size (min)				
<10 mm	9.52 ± 4.63	13.9 ± 2.6 [12–15]	5.52 ± 1.01 [5–6]	<0.001
10–20 mm	4.98 ± 0.77	5.05 ± 0.84 [5–5]	3.89 ± 0.92 [4–5]	0.03
20–30 mm	4.9 ± 0.79	4.7 ± 0.9 [4.25–5]	4.85 ± 0.88 [4–5]	0.513
Segment				
Simple	4.7 ± 0.92	4.72 ± 1.2 [4.25–5]	4.82 ± 0.85 [4–5]	0.53
Complex	6,73 ± 3.66	7.49 ± 4.25 [5–11]	5.11 ± 0.97 [4–6]	<0.001
Visceral pleura distance				
Peripheral	4.9 ± 1.31	5.3 ± 3.4 [5–5]	4.7 ± 0.79 [4–5]	0.11
Deep	7.14 ± 4.22	9.58 ± 4.82 [9–12]	5.4 ± 1.03 [4–6]	<0.001

Abbreviation: IQR, interquartile range; SD, standard deviation.

#### Size of lesion

**FIGURE 3:**
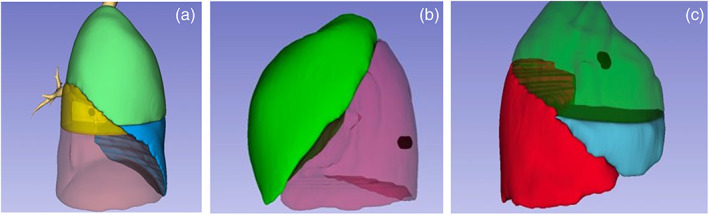
Examples of lesions <10 mm (a); of lesions between 10 and 20 mm (b); and of lesions between 20 and 30 mm (c).

The mean operative time for identification of lesion size less than 10 mm, between 10 and 20 mm, and between 20 and 30 mm were 9.52 ± 4.63 min; 4.98 ± 0.77 min; and 4.9 ± 0.79 min, respectively. The standard group compared to the 3D group was associated with a significantly longer operative time for lesions <10 mm (13.87 ± 2.59 min vs. 5.52 ± 1.01 min, *p* < 0.001, respectively; Figure 3a), and for lesions between 10 and 20 mm (5.05 ± 0.84 min vs. 3.89 ± 0.92 min, *p* = 0.03; Figure 3b), while no significant difference was found for lesions between 20 and 30 mm (4.69 ± 0.89 min vs. 4.85 ± 0.88 min; *p* = 0.513; Figure 3c).

#### Segmental localization

The mean operative time for the detection of lesions localized in a complex segment was longer than that sited in a simple segment (6.73 ± 3.66 min vs. 4.7 ± 0.92 min, *p* < 0.0001). The 3D group compared to the standard group was associated with a lower operative time for the identification of nodules localized in complex segments (7.49 ± 4.25 min vs. 5.11 ± 0.97 min; *p* < 0.001), but no significant difference was found for the detection of nodules localized in simple segments (4.72 ± 1.2 min vs. 4.85 ± 0.85 min; *p* = 0.53).

#### Distance from visceral pleura

**FIGURE 4:**
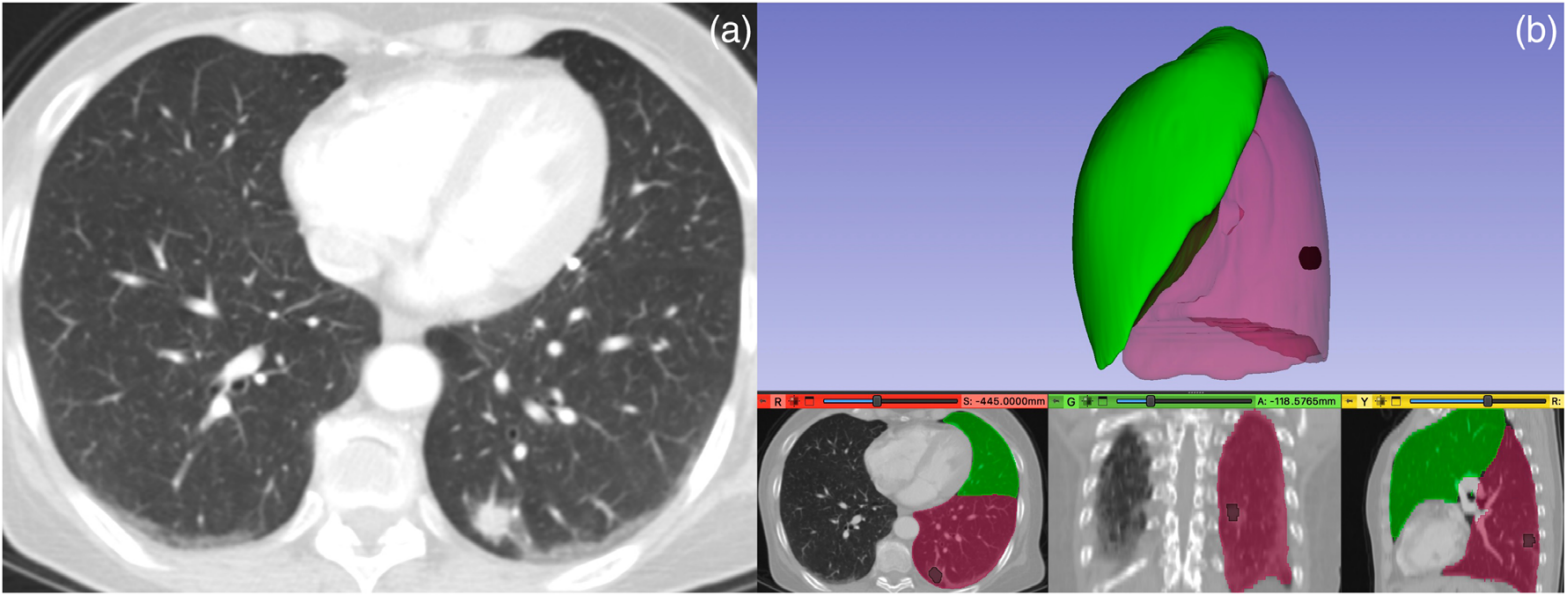
A 63‐year‐old woman with a lung adenocarcinoma. (a) Computed tomography (CT) scan showed a tumor located in the left lower lobe (LLL). (b) Digital 3D model with left upper lobe (LUL) (green), left lower lobe (LLL) in transparency (red) and nodule (black) in LLL. The surgical operation is shown in Video S2.

**FIGURE 5:**
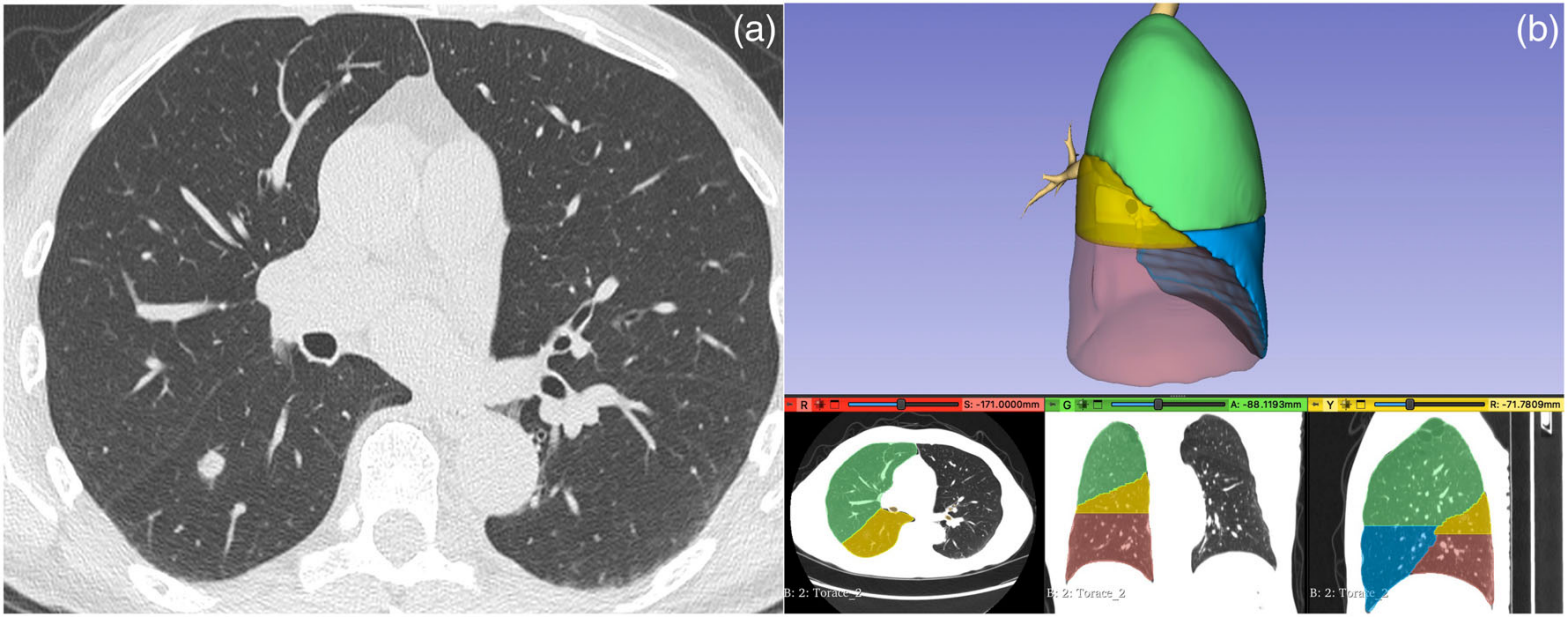
A 61‐year‐old man with a 9 mm adenocarcinoma. (a) Computed tomography (CT) scan showed tumor located in right S6. (b) Digital 3D model with right S6 (yellow) and right lower lobe (RLL) in transparency (red), middle lobe (ML) (azure), right upper lobe (RUL) (green) and nodule (black) in S6.

The mean operative time for the detection of deep lesions was longer than that of peripheral lesions (7.14 ± 4.22 min vs. 4.9 ± 1.31 min, *p* < 0.0001). The 3D group compared to the standard group was associated with a lower operative time for identification of deep nodules (9.58 ± 4.82 min vs. 5.4 ± 1.03 min; *p* < 0.001), but no significant difference was found for peripheral nodules (5.3 ± 3.4 min vs. 4.7 ± 0.79 min; *p* = 0.11). Box plots showing the differences between the two groups are shown in Figures 4 and 5.

## DISCUSSION

The thoracoscopic detection of lung nodules may be challenging especially in cases of deep and small lesions due the impossibility of manual palpation of the lung. Quickly and accurately localizing lesions can prevent prolonged operative time and conversion to thoracotomy. To facilitate the identification of lung nodules during surgery, several techniques have been proposed over the years. Methylene blue staining is the most used method,^2^, ^9^ but it can spread to surrounding tissues and prevent intraoperative nodule recognition. Hook‐wire and micro‐coils overcome this limitation,^10^, ^11^ but they are associated with an increased risk of complications such as pneumothorax and hemoptysis and they cannot be used for lesions close to the heart, large blood vessels, diaphragm and major nerves due to the high risk of injury. Thus, more sophisticated and less invasive strategies have been proposed such as the use of radiotracers and/or fluorescence tracers for marking the nodule in some cases under ultrasound or electromagnetic navigation bronchoscopy.^12^, ^13^ However, these techniques require sophisticated and specific equipment as hybrid operative rooms are not widely available.

Over the last few years, the use of 3D models has increased in thoracic surgery for planning complex surgical procedures and/or as a scaffold to create custom stents not commercially available.^14^, ^15^, ^16^ Recently, Ji et al.^17^ used 3D reconstructions and printed 3D models for the identification of the lung nodule in surgically resected samples.

However, to the best of our knowledge, no study prior to the present study has reported the use of a 3D lung model for guiding localization of lung nodules during surgery. To test the validity of our model, we planned a study where the use of 3D lung model for detecting lung nodules was compared to a control group and the reduction of procedural time was the main outcome. To reduce the intergroup differences due to the lack of standardization, only patients treated after 2019 were included in the study as in our institution we started to perform thoracoscopic anatomical resections from 2019. Furthermore, GGO lesions were excluded as their detection could be more difficult compared to subsolid solid lesions.^18^, ^19^

First, the two study groups showed no significant difference regarding the characteristics of lesions. The use of the 3D model facilitated the localization of lung nodules during surgery as confirmed by the reduction of operating time observed in the 3D group compared to the standard group. As expected, the difference was more evident for deep and small lesions (<10 mm) and localized in complex segments. Conversely, in cases of peripheral and large lesions, or localized in a simple segment, the use of a 3D model did not confer any significant advantages. Peripheral nodules retracted visceral pleural may be visually detected, yet large nodules may be easily palpated especially if they are in accessible segments. These studies likely explain the lack of any advantages observed with the use of the 3D model. Our data were in line with Zhang et al.^13^ who evaluated the use of the 3D model as a guide for the insertion of the hook‐wire in lung nodules and found that this strategy reduced the procedural time and the complication rates only in cases of small nodules less than 20 mm.

Second, our strategy presented several advantages compared to all previous procedures. Specific equipment was not required nor were there any additional health care costs, being open software. Additionally, it was not associated with any complications for the patients being a noninvasive procedure. Furthermore, transferring the 3D model to an operating room monitor facilitated the images to be viewed in real time during the operation and to guide the identification of the lesion. Also, if an additional monitor was not available in an operating room, the 3D model could easily be transferred to a standard mobile device such as a tablet.^16^ On the other hand, our strategy presented several limitations that might limit its use. It was unable to visually mark the target nodule as for other tracers. Thus, the surgeon should identify the nodule based on the information obtained by the 3D model as the exact position of the nodule within the segment and/or the relation of the nodule with respect to other anatomical landmarks as the visceral pleura or the fissures. The CT scan images should be defined characteristics for creation of the digital 3D model. The model creation was a time‐consuming procedure. The current segmentation process was manual and performed on a 3D slicer prior to the procedure. The operator identified the region of interests (i.e., nodule, lobes, segments) and based on the different density of the structures, the software created separate models that allow the target lesion to be separated from the other structures. It was a semiautomatic process as the operator could define the target lesions to reconstruct and then differentiate. The next step of our program will be to create software that automatically show each structure with a different color based on radiological density without additional actions by the operator. It may reduce the time for 3D lung model creation and facilitate its application.

Our study had several limitations and no definitive conclusions can therefore be drawn. (1) Selection bias due to the lack of randomization was the main limitation. (2) Specific anatomical variants or the presence of emphysema could make identification of the nodule difficult, but this was not evaluated in this study. (3) All procedures were performed by one surgeon (AF), thus the lack of interobserver agreement by two surgeons could affect the study results.

In conclusion, our 3D model is an additional tool for thoracic surgeons to guide the detection of small and deep nodules during thoracoscopy. It is a noninvasive and cost saving procedure and may be widely used. In addition, 3D reconstructions can have a didactic utility whereby it is possible to integrate them into the training of young surgeons. Due to the retrospective nature of the study, our results should be corroborated by randomized and larger studies.

## CONFLICT OF INTEREST STATEMENT

The authors declare no conflicts of interest.

## Supporting information


**Video S1.** Digital 3D model transferred to a mobile device.

**Video S2.** The digital 3D model guided in real time the localization of lung nodule during surgery.

## Data Availability

The data underlying this article are available here and in the online supplementary material.
